# Centres for Healthcare Improvement: solution to the quality problem

**DOI:** 10.1258/jrsm.2011.110076

**Published:** 2011-07

**Authors:** Julie E Reed, Martin Marshall, Derek Bell

**Affiliations:** 1 NIHR CLAHRC for Northwest London, Imperial College, Chelsea and Westminster Hospital NHS Foundation Trust, London, UK; 2 Health Foundation, London, UK

Few healthcare systems are exempt from a seemingly constant cycle of reform and this is particularly apparent in the USA and England at present. In part this is driven by the need to provide more cost-effective care, and hence savings, but also by an increased awareness of variable quality of care for patients.^1,2^ Even so, large-scale reorganization and reform is often undertaken with little thought given to how changes will be implemented at a local level.^3^ The consequence is that often we see a ‘voltage drop’ – sometimes a significant one – between policy planners and implementation in the front line. The ‘doing’ always gets forgotten.

The complexity and context-dependent nature of delivering improvement is well-recognized. Research has demonstrated that the skills and expertise needed to successfully deliver change include effective leadership, engagement of staff and stakeholders, and alignment with strategic goals.^4,5^ Continuing to identify the same solutions or problems in different settings can no longer be considered added value research. If we are to deliver the recent Health and Social Care Bill's^6^ commitment to continuous improvement in healthcare we must develop local infrastructures to embed an evidence-based systematic approach to ‘doing’. Thus supporting the implementation of policy and research to improve everyday care.

A potential solution is to create local Centres for Healthcare Improvement serving a defined health economy. The primary function of these Centres would be to work within the local health economy to deliver tangible and sustained improvements at the point of care. They would provide expert advice and directly support the delivery of national and local priorities through utilizing real-world research and improvement science to inform and drive ‘doing’. Aspects of this work could be undertaken as a form of internal consultancy to support more cost-effective systems of care.

Second, the Centres would provide an integrated network for collaboration within the local health and research community, engaging all healthcare organizations, higher education institutes, the local community and patients plus relevant industry partners within the network. Through the network partner organizations would share expertise in research, improvement science and leadership to coordinate the delivery of higher quality care. The network would thus provide a mechanism for peer-support and challenge, through shared learning, to increase effectiveness and reduce duplication. Centres would share learning with each other and provide opportunities for local, national and international networking.

Third, to sustain improvements and maximize effectiveness the proposed Centres would build staff capacity and capability across the interface between the NHS and academia, recognizing that managers, clinicians and frontline staff are key actors in affecting large-scale change and implementing evidence-based medicine.^4,7^ The Centres would develop staff with the core knowledge and skills and create a permissive culture for experiential practice – learning through ‘doing’– combined with formal education for healthcare professionals (undergraduate, postgraduate or practice-based).

To support the NHS, academic researchers require training to increase their awareness of the contextual issues and day-to-day challenges faced by staff working at the front line to ensure research is relevant and practical. Overcoming current methodological divides will be essential to ensure results are meaningful and delivered in a timely fashion to support quality-driven change in the NHS.

Ultimately, it is crucial that a systematic and scientific approach to ‘doing’ is developed to support utilization of best evidence, creation and utilization of new knowledge and to underpin new developments with continuous evaluation. Failure to do this will mean that we repeat the same historical mistakes, i.e. not translating knowledge into action and delivery. By providing a central resource for healthcare research and improvement, the Centres would act as a ‘translator’ and ‘communicator’ across traditionally disparate groups. Currently these groups are distinguished by different languages and epistemological approaches and with different goals and incentives. In the future these groups must work collaboratively and creatively to develop the emerging academic field of improvement science,^8^ using rigorous and pragmatic methodologies that can reliably drive change for patient benefit at the front line.

All health economies would benefit from a Centre for Healthcare Improvement. Their development may be limited by the presence or willingness of local academic and NHS organizations with the knowledge, skills and commitment of all parties to work together. Nevertheless, we believe that there is capacity and capability to establish at least 10 Centres around the UK in the next five years.

All parties responsible for health, research and delivery must acknowledge the need for a systematic and evidence-based approach to the delivery of sustainable improvements in care. This will require resource (currently 1% of funding is spent on implementation research).^9^ The strategy Best Research for Best Health emphasized the need for health research to deliver tangible improvements. This led to the development of a NHS-centred research infrastructure (including Biomedical Research Centres and Units and Collaborations for Leadership in Applied Health Research and Care). Greater integration of these organizations with providers and commissioners will be necessary to ensure research outputs are more closely linked to real improvements in patient care. Centres for Healthcare Improvement could provide the mechanism to deliver this.

## DECLARATIONS

### Competing interests

None declared

### Funding

None

### Ethical approval

Not applicable

### Guarantor

DB

### Contributorship

All authors contributed equally

### Acknowledgements

None
